# Isolation, characterization, and pathogenicity of *Fusarium* species causing crown rot of wheat

**DOI:** 10.3389/fmicb.2024.1405115

**Published:** 2024-05-30

**Authors:** Guoping Ma, Heng Wang, Kai Qi, Liguo Ma, Bo Zhang, Yueli Zhang, Hang Jiang, Xuehong Wu, Junshan Qi

**Affiliations:** ^1^Institute of Plant Protection, Shandong Academy of Agricultural Sciences, Shandong Key Laboratory of Plant Virology, Jinan, China; ^2^College of Life Sciences, Shandong Normal University, Jinan, China; ^3^Department of Plant Pathology, China Agricultural University, Beijing, China

**Keywords:** wheat, *Fusarium* crown rot, *Fusarium* spp., characterization, pathogenicity

## Abstract

Fusarium crown rot (FCR) is one of the most important soilborne diseases affecting wheat production. To investigate the diversity of the pathogens causing this disease, 199 diseased wheat samples were collected from 13 cities in Shandong province. In total, 468 isolates were obtained, and from these isolates, 11 *Fusarium* species were identified based on phylogenetic analyses with the translation elongation factor-1α (*TEF-1α*), RNA polymerase II largest subunit (*RPB1*), and RNA polymerase II second largest subunit (*RPB2*) gene sequences. Of these *Fusarium* isolates, 283 were identified as *Fusarium pseudograminearum* and the remaining isolates were identified as *Fusarium graminearum* (*n* = 113), *Fusarium sinensis* (*n* = 28), *Fusarium acuminatum* (*n* = 18), *Fusarium incarnatum* (*n* = 13), *Fusarium ipomoeae* (*n* = 5), *Fusarium flocciferum* (*n* = 3), *Fusarium proliferatum* (*n* = 2), *Fusarium asiaticum* (*n* = 1), *Fusarium culmorum* (*n* = 1), and *Fusarium oxysporum* (*n* = 1), suggesting that *F. pseudograminearum* is the dominant pathogen of FCR of wheat in Shandong province. Pathogenicity tests demonstrated that all 11 *Fusarium* species could cause typical symptoms of FCR on wheat seedlings. The results of the study indicate that a greater diversity of *Fusarium* species can cause FCR of wheat in Shandong province than that has been previously reported. This is the first report in the world of *Fusarium incarnatum*, *Fusarium ipomoeae*, and *Fusarium flocciferum* as pathogens causing FCR in wheat.

## Introduction

Wheat (*Triticum aestivum* L.) is the second most important grain crop and is grown in diverse areas worldwide (Singh et al., 2016). Fusarium crown rot (FCR) of wheat is one of the most destructive soil−/residue-borne diseases in many arid and semi-arid cropping regions of the world (Kazan and Gardiner, 2018). This disease was causing damage to the wheat plant in China but only in a limited way before 2010. In recent years, it has become highly prevalent in the Huanghuai wheat-growing area, in part due to the adoption of moisture-preserving cultural practices, such as minimum tillage and stubble retention (Deng et al., 2020).

FCR occurs in the seedling stage, causing the death of seedlings before or after emergence. Brown discoloration appears on the coleoptile, subcrown internode, lower leaf sheaths and adjacent stems, and nodal tissues of the survived seedlings. The browning of the lower stems occurs with an occasional pink coloration of the nodes or stems under the leaf sheaths (Kazan and Gardiner, 2018). This disease process culminates with premature senescence of heads, called whiteheads, with no or shriveled grains (Kazan and Gardiner, 2018; Zhou et al., 2019). The incidence of FCR and its severity are negatively correlated with grain yield, tiller height, and straw weight (Smiley et al., 2005). Smiley et al. (2005) reported that FCR can cause up to 35% reduction in wheat grain yield under natural inoculum in the Pacific Northwest of the United States. In addition, FCR may lead to the contamination of wheat grains by mycotoxins (Mudge et al., 2006).

FCR of wheat is caused by a number of *Fusarium* species, and the composition of *Fusarium* species varies among regions. In the UK, *Fusarium avenaceum* and *Fusarium culmorum* were the pathogens causing FCR (Pettitt et al., 2003). In Queensland and northern New South Wales, *Fusarium acuminatum*, *Fusarium avenaceum*, *Fusarium babinda*, *Fusarium crookwellense*, *Fusarium graminearum*, *Fusarium subglutinans*, *Fusarium torulosum*, *Fusarium tricinctum*, *Fusarium proliferatum*, and *Fusarium pseudograminearum* were aggressive in causing FCR (Akinsanmi et al., 2004). In Turkey, six *Fusarium* species, such as *F. avenaceum*, *F. culmorum*, *F. graminearum*, *Fusarium hostae*, *F. pseudograminearum*, and *Fusarium redolens*, could cause crown rot with different levels of severity (Shikur Gebremariam et al., 2018). Among 12 *Fusarium* species isolated from diseased wheat samples in Azerbaijan, *Fusarium algeriense*, *F. avenaceum*, *F. culmorum*, *F. graminearum*, *F. hostae*, and *F. pseudograminearum* were pathogenic to wheat (Özer et al., 2020).

With ongoing research, a greater number of *Fusarium* species have been identified to cause FCR in a certain wheat-growing area. In China, a previous survey on agents causing FCR in Anhui, Jiangsu, Henan, Shandong, and Hebei provinces revealed that *F. acuminatum*, *F. asiaticum*, *F. avenaceum*, *F. graminearum*, and *F. pseudograminearum* were the pathogens responsible for the disease (Zhang et al., 2015). *F. acuminatum*, *F. asiaticum*, *F. culmorum*, *F. equiseti*, *F. graminearum*, *F. oxysporum*, *F. proliferatum*, *F. pseudograminearum*, and *F. sinensis* were the pathogens causing FCR in the Huanghuai wheat-growing region (including Anhui, Jiangsu, Henan, Shanxi, Shaanxi, Shandong, and Hebei provinces) (Zhou et al., 2019).

Information on species complexity is essential for designing effective management strategies, especially since different species of *Fusarium* exhibit varying degrees of sensitivity to fungicides. Previous research reported that *Fusarium verticillioides* was sensitive to tebuconazole, with inhibition values of 94%, while *F. proliferatum* and *F. graminearum* showed lower inhibition values of 77 and 67%, respectively (Masiello et al., 2019). Therefore, the objectives of this study were to isolate and identify the *Fusarium* species causing FCR of wheat in Shandong province and evaluate the pathogenic diversity of different *Fusarium* species on wheat seedlings so that suitable strategies could be developed for disease management.

## Materials and methods

### Sample collection and *Fusarium* isolation

**Figure 1:**
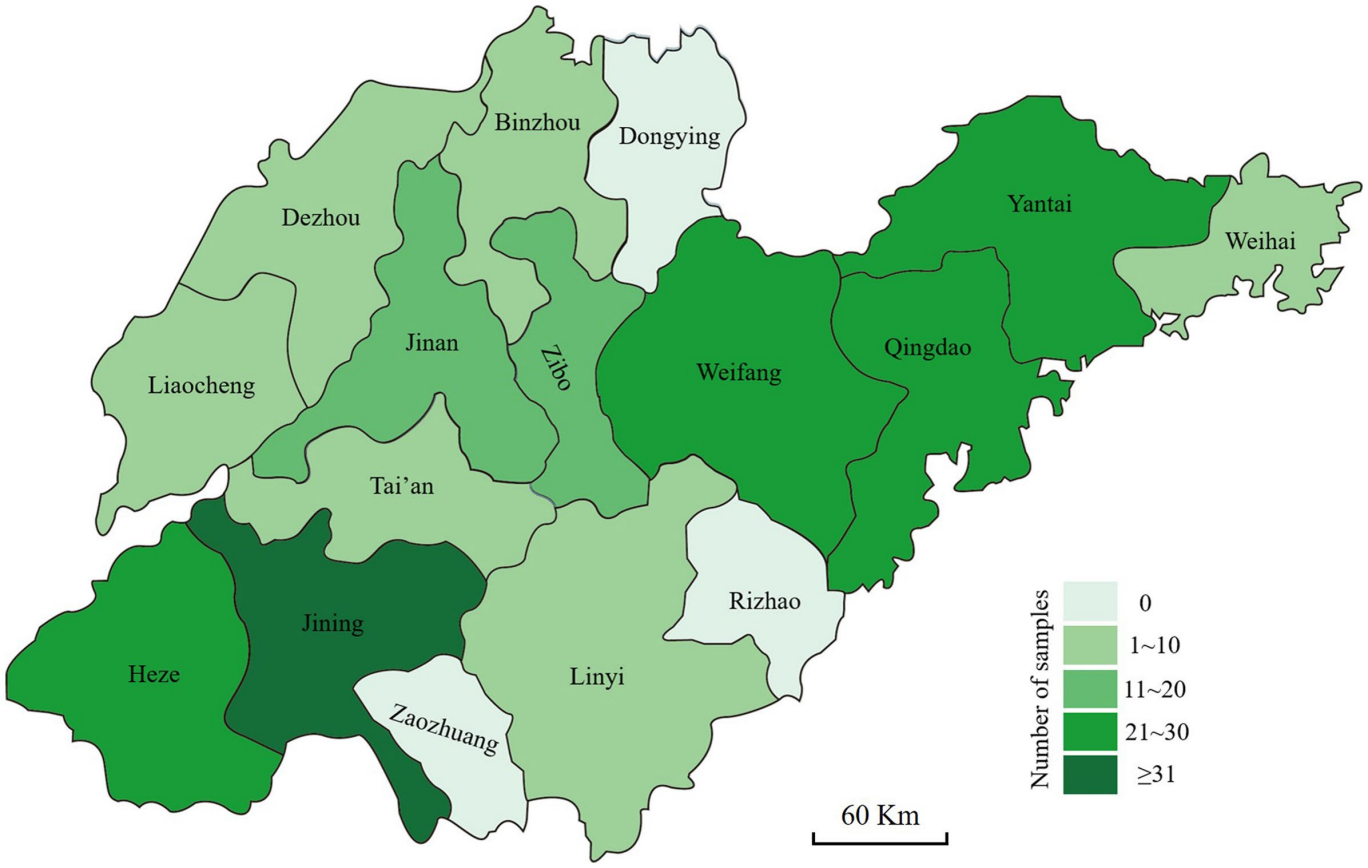
Geographic origins where the diseased wheat samples with symptoms of *Fusarium* crown rot were collected in Shandong province.

The stems of the diseased wheat plants exhibiting crown rot symptoms were collected from Shandong province. The wheat fields were selected randomly, and the selected fields were at least 3 km apart. The area of each field was more than 667 m^2^. At least six wheat fields in each city were selected for sample collection. The samples were collected from five sites in the field in a zigzag pattern (Fang, 1998). Each sampling site was approximately 1 m^2^ and at least 10 m apart. Three diseased wheat plants were collected from each sampling site, meaning that one sample consisted of 15 diseased plants. In total, 199 samples were collected from 13 cities (Figure 1). Small tissue pieces (approximately 3–6 mm in length) were cut from healthy to diseased margins, surface-sterilized with 70% ethanol for 40 s and 0.5% sodium hypochlorite (NaClO) solution for 2 min, rinsed with sterilized water three times, and then air dried on sterilized filter papers. The pieces were placed on potato dextrose agar (PDA) (Abate et al., 2018) plates containing 50 μg/mL streptomycin sulfate and incubated at 25°C in the dark for 48–72 h. Suspected *Fusarium* colonies were transferred to fresh PDA plates, and pure cultures were obtained from hyphal tips. Then, the *Fusarium*-like isolates were obtained and stored at −4°C for further studies.

### DNA extraction and polymerase chain reaction (PCR) amplification

All the *Fusarium*-like isolates were grown on PDA plates for 4–7 days at 25°C in the dark. A sample of the mycelia (20 mg) of each isolate was carefully collected from the agar medium surface and ground to a fine powder in liquid nitrogen. Genomic DNA was extracted using the cetyltrimethylammonium bromide (CTAB) method, as described by Lee and Taylor (1990). The obtained DNA pellet was dried under vacuum, dissolved in 30 μL ddH_2_O, and stored at −20°C until use.

The partial translation elongation factor-1α (*TEF-1α*), RNA polymerase II largest subunit (*RPB1*), and RNA polymerase II second largest subunit (*RPB2*) genes were amplified with the primers EF1 and EF2 (O’Donnell et al., 1998), F7 and G2R (O’Donnell et al., 2022), and 5f2 and 7cr (O’Donnell et al., 2022) (Table 1). The PCR reaction mixture consisted of 10.5 μL ddH_2_O, 12.5 μL 2× F8 FastLong PCR MasterMix (PC80, Aidlab Biotechnologies Co., Ltd., Beijing, China; containing 0.05 units/μL F8 FastLong DNA Polymerase, 0.4 mM dNTPs, and 4 mM MgCl_2_), 0.5 μL of each primer (10 μM), and 1 μL DNA template (100 μg/mL). Negative controls contained the same reagents but without the DNA template. Amplifications were performed in an Eppendorf Mastercycler gradient thermal cycler (Eppendorf, Hamburg, Germany). All primers and PCR conditions are summarized in Table 1.

**Table 1:** Amplification sites, primer names, primer sequences, PCR conditions, and references used in this study.

**Locus**	**Primer name**	**Primer Sequence (5′-3′)**	**PCR conditions**	**References**
*TEF-1α*	EF1	ATGGGTAAGGARGACAAGAC	94°C for 3 min (94°C for 10 s, 53°C for 15 s, and 72°C for 8 s) × 35 cycles, 72°C for 5 min	O’Donnell et al. (1998)
	EF2	GGARGTACCAGTSATCATGTT		
*RPB1*	F7	CRACACAGAAGAGTTTGAAGG	94°C for 3 min (94°C for 10 s, 53°C for 15 s, and 72°C for 11 s) × 35 cycles, 72°C for 5 min	O’Donnell et al. (2022)
	G2R	GTCATYTGDGTDGCDGGYTCDCC		
*RPB2*	5f2	GGGGWGAYCAGAAGAAGGC	94°C for 3 min (94°C for 10 s, 54°C for 15 s, and 72°C for 11 s) × 35 cycles, 72°C for 5 min	O’Donnell et al. (2022)
	7cr	CCCATRGCTTGYTTRCCCAT		

### DNA sequencing and phylogenetic analysis

The PCR products were purified with an Aidlab DNA Gel Extraction Kit (Aidlab Biotechnologies) and cloned into a pTOPO-T Simple Vector (CV15, Zero Background pTOPO-TA Simple Cloning Kit, Aidlab Biotechnologies) according to manufacturer’s instructions. The ligation reaction mixture was transformed into competent cells of *Escherichia coli* TreliefTM 5α (TSC-C01, Qingdao Tsingke Biotechnology Co., Ltd., Qingdao, China), and transformants were cultured on Luria-Bertani (LB) agar plates containing ampicillin (50 μg/mL), 5-bromo-4-chloro-3-indolyl-β-d-galactoside (X-gal, 100 μg/mL), and isopropyl-b-d-thiogalactopyranoside (IPTG, 100 μg/mL). White colonies with the target DNA insertion verified by PCR were sent to Qingdao Tsingke Biotechnology for sequencing.

All the isolates were initially examined molecularly by the sequence analysis of the *TEF-1α* gene. The putative identifications were made based on the percent shared identity of consensus sequences to related *Fusarium* species in the NCBI GenBank database using BLASTn searches. To further verify the accuracy of characterization using the *TEF-1α* gene, 53 isolates representing 11 different *Fusarium* species according to the *TEF-1α* gene sequence analysis were then examined for the *RPB1* and *RPB2* gene regions. Multiple sequence alignments were constructed using an online version of MAFFT v.7 (https://mafft.cbrc.jp/alignment/server/) (Katoh and Standley, 2013). The aligned sequences were edited using BioEdit software and completed by manual adjustments. The maximum likelihood (ML) analyses of independent (*TEF-1α*) and concatenated (*TEF-1α*, *RPB1*, and *RPB2*) gene datasets were performed using RAxML-HPC BlackBox v.8.2.10 (Stamatakis, 2006) within the Cyberinfrastructure for Phylogenetic Research (CIPRES) portal (https://www.phylo.org/portal2/) (Miller et al., 2010). Branch stability was estimated with 1,000 bootstrap replicates. Sequences of *Stemphylium vesicarium* or *Fusarium solani* served as the outgroup taxon in the analyses. The phylogenetic trees were viewed in MEGA v.7.0, and bootstrap values ≥70% were considered as significant and indicated in the phylogenetic trees. The basic information of 53 representative isolates in this study, 22 representative isolates of the 11 *Fusarium* species, and outgroup *S. vesicarium* strain CBS 191.86 and two strains of *F. solani* (NRRL 23244 and 32,810) are shown in Table 2.

**Table 2:** Isolates included in the phylogenetic analysis and their GenBank accession numbers.

**Isolate**	**Species**	**Location**	**GenBank accession no.**		
			***TEF-1α***	***RPB1***	***RPB2***
BZ3-1	*Fusarium pseudograminearum*	Binzhou, Shandong	OP105166	OP785174	OP785227
BZ4-2	*F. pseudograminearum*	Binzhou, Shandong	OP105167	OP785175	OP785228
DLY1-1	*F. pseudograminearum*	Dezhou, Shandong	OP105168	OP785176	OP785229
DN2-1	*F. pseudograminearum*	Dezhou, Shandong	OP105169	OP785177	OP785230
DY1-2	*F. pseudograminearum*	Dezhou, Shandong	OP105170	OP785178	OP785231
HD1-1	*F. pseudograminearum*	Heze, Shandong	OP105171	OP785179	OP785232
HJ2-2	*F. pseudograminearum*	Heze, Shandong	OP105172	OP785180	OP785233
HM4-1	*F. pseudograminearum*	Heze, Shandong	OP105173	OP785181	OP785234
HY2-4	*F. pseudograminearum*	Heze, Shandong	OP105174	OP785182	OP785235
JJ1-1	*F. pseudograminearum*	Jining, Shandong	OP105175	OP785183	OP785236
JJ7-2	*F. pseudograminearum*	Jining, Shandong	OP105176	OP785184	OP785237
JL1-1	*F. pseudograminearum*	Jining, Shandong	OP105177	OP785185	OP785238
JL14-1	*F. pseudograminearum*	Jining, Shandong	OP105178	OP785186	OP785239
JT1-1	*F. pseudograminearum*	Jinan, Shandong	OP105179	OP785187	OP785240
JW1-1	*F. pseudograminearum*	Jining, Shandong	OP105180	OP785188	OP785241
LC1-1	*F. pseudograminearum*	Liaocheng, Shandong	OP105181	OP785189	OP785242
LG1-2	*F. pseudograminearum*	Liaocheng, Shandong	OP105182	OP785190	OP785243
LJ1-1	*F. pseudograminearum*	Linyi, Shandong	OP105183	OP785191	OP785244
LY1-1	*F. pseudograminearum*	Linyi, Shandong	OP105184	OP785192	OP785245
QL2-1	*F. pseudograminearum*	Qingdao, Shandong	OP105185	OP785193	OP785246
QP1-1	*F. pseudograminearum*	Qingdao, Shandong	OP105186	OP785194	OP785247
QP3-3	*F. pseudograminearum*	Qingdao, Shandong	OP105187	OP785195	OP785248
TF1-1	*F. pseudograminearum*	Tai’an, Shandong	OP105188	OP785196	OP785249
WB1-1	*F. pseudograminearum*	Weifang, Shandong	OP105189	OP785197	OP785250
WC1-3	*F. pseudograminearum*	Weifang, Shandong	OP105190	OP785198	OP785251
WC9-2	*F. pseudograminearum*	Weifang, Shandong	OP105191	OP785199	OP785252
WR5-1	*F. pseudograminearum*	Weihai, Shandong	OP105192	OP785200	OP785253
YL4-1	*F. pseudograminearum*	Yantai, Shandong	OP105193	OP785201	OP785254
ZH2-1	*F. pseudograminearum*	Zibo, Shandong	OP105194	OP785202	OP785255
BH1-2	*F. graminearum*	Binzhou, Shandong	OP105195	OP785203	OP785256
DN2-3	*F. graminearum*	Dezhou, Shandong	OP105196	OP785204	OP785257
HJ1-1	*F. graminearum*	Heze, Shandong	OP105197	OP785205	OP785258
JJ2-1	*F. graminearum*	Jining, Shandong	OP105198	OP785206	OP785259
JL10-1	*F. graminearum*	Jining, Shandong	OP105199	OP785207	OP785260
JT3-2	*F. graminearum*	Jinan, Shandong	OP105200	OP785208	OP785261
QC1-1	*F. graminearum*	Qingdao, Shandong	OP105201	OP785209	OP785262
TF1-4	*F. graminearum*	Tai’an, Shandong	OP105202	OP785210	OP785263
WR3-1	*F. graminearum*	Weihai, Shandong	OP105203	OP785211	OP785264
YLY2-1	*F. graminearum*	Yantai, Shandong	OP105204	OP785212	OP785265
ZL3-2	*F. graminearum*	Zibo, Shandong	OP105205	OP785213	OP785266
DP1-1	*F. sinensis*	Dezhou, Shandong	OP105206	OP785214	OP785267
HY6-1	*F. sinensis*	Heze, Shandong	OP105207	OP785215	OP785268
WS2-3	*F. sinensis*	Weifang, Shandong	OP105208	OP785216	OP785269
LS1-1	*F. acuminatum*	Liaocheng, Shandong	OP105209	OP785217	OP785270
YZ1-3	*F. acuminatum*	Yantai, Shandong	OP105210	OP785218	OP785271
JJ6-2	*F. incarnatum*	Jining, Shandong	OP105211	OP785219	OP785272
ZL2-1	*F. incarnatum*	Zibo, Shandong	OP105212	OP785220	OP785273
JWS1-1	*F. ipomoeae*	Jining, Shandong	OP105213	OP785221	OP785274
QL1-1	*F. flocciferum*	Qingdao, Shandong	OP105214	OP785222	OP785275
WC1-5	*F. proliferatum*	Weifang, Shandong	OP105215	OP785223	OP785276
WR3-2	*F. asiaticum*	Weihai, Shandong	OP105216	OP785224	OP785277
LS1-3	*F. culmorum*	Liaocheng, Shandong	OP105217	OP785225	OP785278
JL3-3	*F. oxysporum*	Jining, Shandong	OP105218	OP785226	OP785279
NRRL 28062	*F. pseudograminearum*	— ^a^	**AF212468**	**JX171524**	**JX171637**
NRRL 28065	*F. pseudograminearum*	—	**AF212469**	**MG282389**	**MG282419**
NRRL 31084	*F. graminearum*	—	**MW233103**	**JX171531**	**JX171644**
NRRL 52929	*F. graminearum*	—	**JF740871**	**JF741018**	**JF741196**
CBS 122710	*F. sinensis*	—	**EF531235**	—	—
CBS 122711	*F. sinensis*	—	**EF531238**	—	—
NRRL 13332	*F. acuminatum*	—	**OL772797**	**OL772949**	**OL773101**
NRRL 13406	*F. acuminatum*	—	**OL772805**	**OL772957**	**OL773109**
NRRL 13379	*F. incarnatum*	—	**GQ505591**	—	**GQ505769**
NRRL 32866	*F. incarnatum*	—	**GQ505615**	**HM347162**	**GQ505793**
NRRL 43640	*F. ipomoeae*	—	**GQ505667**	**HM347191**	**GQ505845**
NRRL 45996	*F. ipomoeae*	—	**GQ505671**	**KC808326**	**GQ505849**
NRRL 40008	*F. flocciferum*	—	**OL772897**	**OL773049**	**OL773201**
NRRL 45999	*F. flocciferum*	—	**OL772882**	**OL773034**	**OL773186**
NRRL 62905	*F. proliferatum*	—	**MN193865**	**MN193921**	**MN193893**
NRRL 66289	*F. proliferatum*	—	—	**MG282386**	**MG282416**
NRRL 13818	*F. asiaticum*	—	**MW233069**	**MW233240**	**MW233412**
NRRL 28720	*F. asiaticum*	—	**AF212453**	—	—
NRRL 25475	*F. culmorum*	—	**AF212463**	**JX171515**	**JX171628**
NRRL 52792	*F. culmorum*	—	**JF740860**	**JF741012**	**JF741186**
NRRL 25378	*F. oxysporum*	—	**HM347116**	**HM347142**	**HM347208**
NRRL 25387	*F. oxysporum*	—	**HM347117**	**HM347143**	**HM347209**
CBS 191.86	*Stemphylium vesicarium*	India	**KC584731**	—	**KC584471**
NRRL 23244	*F. solani*	India	**DQ247568**	—	—
NRRL 32810	*F. solani*	America	**DQ247118**	—	—

^a^“—”: Locations or GenBank accession no. are not available in other studies.

Sequences from GenBank used in the phylogenetic analysis are indicated in bold.

### Pathogenicity tests

Based on the pathogen identification results, 418 representative *Fusarium* isolates, obtained from different cities or counties and representing different *Fusarium* species, were selected to determine the pathogenicity. The experiment was performed with minor modifications of a method described by Zhang et al. (2015). Briefly, tests were conducted on the ‘Jimai 22’ variety of wheat seedlings, and the length of seedlings were approximately 3 cm after pre-germination at 28°C for 3 days. The selected *Fusarium* isolates were incubated on PDA plates at 25°C in the dark for 4 days, and agar plugs (5 mm in diameter) were cut from the edge of the colonies. Ten wheat seedlings were equably arranged on the absorbent gauze strip (approximately 20 × 3 cm [length × width]), and one agar plug was inoculated at the base of each wheat seedling stem. The absorbent gauze strip was then rolled up and placed vertically in an empty Petri dish. Sterile water was added to the dish to keep the gauze moist. The controls consisted of seedlings that were inoculated with sterile plugs of PDA. The dishes were placed in plastic boxes, covered with clear plastic to maintain high humidity, and incubated in a growth chamber at 25°C and 90% relative humidity (RH) with a 12 h photoperiod per day for 7 days. After incubation, disease severity (DS) was scored on a six-point rating system modified from Smiley et al. (2005): 0 = apparently healthy plant with no discoloration of any tissue; 1 = browning of the coleoptile and the browning area < 50%; 2 = browning of the coleoptile and the browning area of 50 ~ 100%; 3 = the browning area exceeded the coleoptile from bottom to top, but the euphylla are still green; 4 = the browning area exceeded the coleoptile from bottom to top, and the euphylla appear to have partial chlorosis; and 5 = whole plant turns yellow or withered and died. Disease index (DI) was calculated using the following formula: DI = [100 × ∑ (*n* × corresponding DS)]/(*N* × 5), where *n* is the number of the infected seedlings corresponding to each disease rating, and *N* is the total number of inoculation seedlings. Re-isolations from the inoculated seedlings were attempted, and the resulting isolates were confirmed as the corresponding *Fusarium* species based on the molecular characteristics described above to fulfill Koch’s postulates. The experiment was conducted three times. Statistical significance was determined with SPSS (v. 20.0; SPSS Inc.) using a least significant difference (LSD) test at a significance level of *P* of <0.05.

## Results

### Fungal isolation and PCR identification

A total of 199 FCR samples resulted in the isolation of a total of 468 *Fusarium* isolates (Table 3). The *TEF-1α* partial gene from all 468 isolates were amplified and sequenced to confirm their identities. The *RPB1* and *RPB2* gene sequences of 53 representative isolates were also analyzed. The basic local alignment search tool (BLASTn) searches using *TEF-1α* partial gene sequence of each isolate showed that all 468 isolates represented the 11 species of *F. pseudograminearum*, *F. graminearum*, *F. sinensis*, *F. acuminatum*, *F. incarnatum*, *F. ipomoeae*, *F. flocciferum*, *F. proliferatum*, *F. asiaticum*, *F. culmorum*, and *F. oxysporum*. This represented an isolate ratio of 60.47, 24.15, 5.98, 3.85, 2.78, 1.07, 0.64, 0.43, 0.21, 0.21, and 0.21%, respectively (Table 3).

**Table 3:** Information of collected isolates from FCR of wheat in Shandong province in this study.

**Geographic origins**	**Number of *Fusarium* isolates** ^**b**^										
	**Fpg**	**Fg**	**Fsi**	**Fac**	**Fi**	**Fip**	**Ff**	**Fpr**	**Fas**	**Fc**	**Fox**
Binzhou	8	2	2	0	1	0	0	0	0	0	0
Dezhou	20	10	1	2	0	0	0	0	0	0	0
Heze	32	14	8	2	1	0	1	0	0	0	0
Jinan	8	13	6	0	0	0	0	1	0	0	0
Jining	59	31	3	1	5	4	0	0	0	0	1
Liaocheng	18	3	0	2	0	0	0	0	0	1	0
Linyi	4	0	0	0	0	0	0	0	0	0	0
Qingdao	23	15	1	2	2	0	2	0	0	0	0
Tai’an	5	3	0	0	0	0	0	0	0	0	0
Weifang	59	4	2	0	0	1	0	1	0	0	0
Weihai	5	4	0	0	0	0	0	0	1	0	0
Yantai	20	9	0	8	2	0	0	0	0	0	0
Zibo	22	5	5	1	2	0	0	0	0	0	0
Total	283	113	28	18	13	5	3	2	1	1	1
Percentage	60.47%	24.15%	5.98%	3.85%	2.78%	1.07%	0.64%	0.43%	0.21%	0.21%	0.21%

^b^Fpg (*F. pseudograminearum*), Fg (*F. graminearum*), Fsi (*F. sinensis*), Fac (F. acuminatum), Fi (F. incarnatum).

Fip (*F. ipomoeae*), Ff (*F. flocciferum*), Fpr (*F. proliferatum*), Fas (*F. asiaticum*), Fc (*F. culmorum*), Fox (*F. oxysporum*).

Of the analyzed wheat samples, 83.42% were infected by individual *Fusarium* species, including 43.72% of the samples infected by *F. pseudograminearum*, 23.12% infected by *F. graminearum*, 9.05% infected by *F. sinensis*, 3.02% infected by *F. acuminatum*, 2.51% infected by *F. incarnatum*, and 0.50% infected by *F. ipomoeae*, *F. flocciferum*, *F. asiaticum*, and *F. culmorum*, respectively; two or three *Fusarium* species were found in 16.58% of the samples isolated from the diseased tissues, and *F. pseudograminearum* or *F. graminearum* combined with other *Fusarium* species infected the vast majority of the samples (Supplementary Table S1).

### Phylogenetic analysis

**Figure 2:**
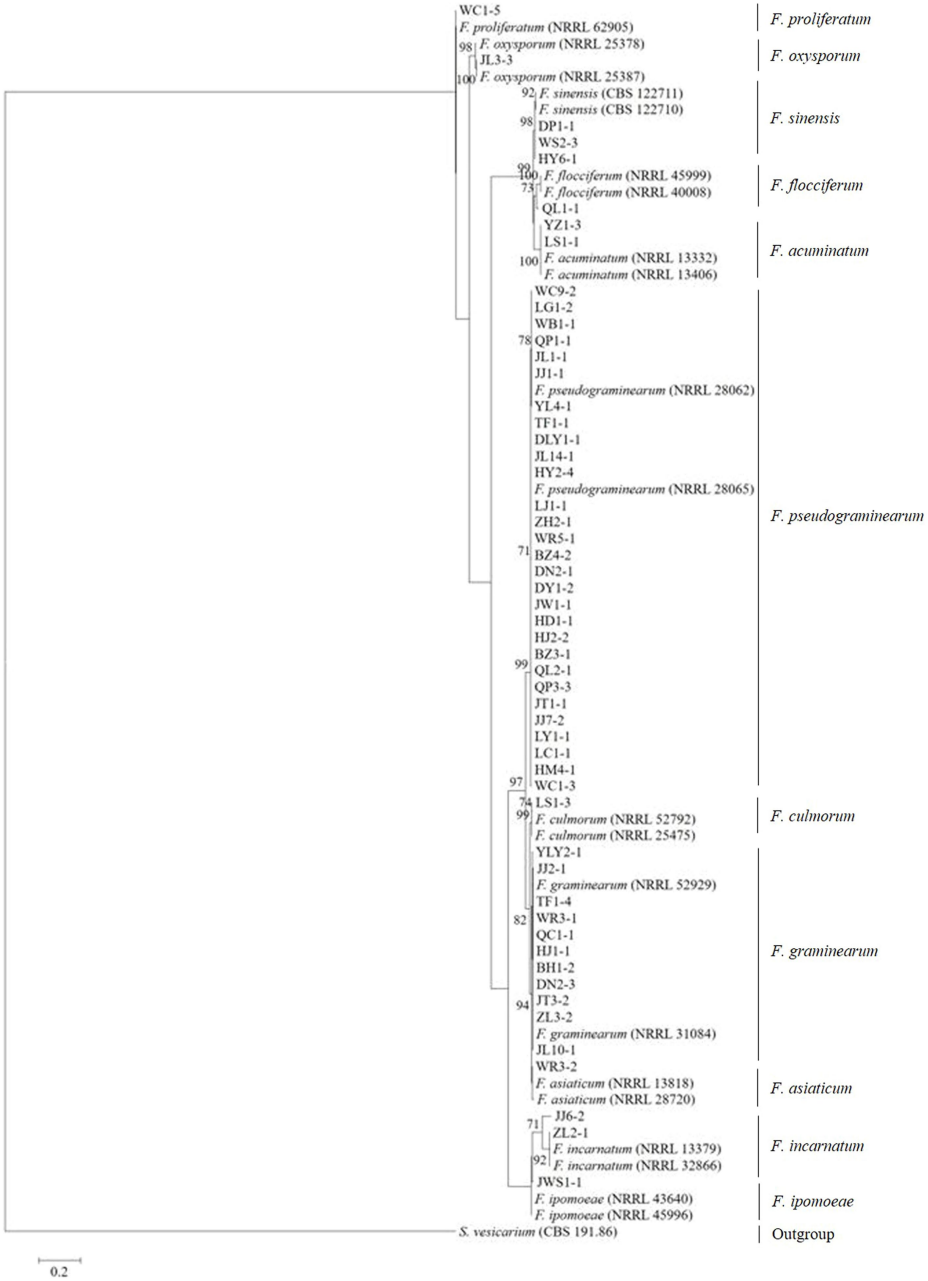
Maximum likelihood phylogenetic analysis of 11 *Fusarium* species based on *TEF-1α* partial gene sequences. The tree was rooted with sequences of *Stemphylium vesicarium*. The number of bootstrap replications was set to 1,000. Support values at nodes represent bootstrap percentages with values ≥70% are shown above the branches.

**Figure 3:**
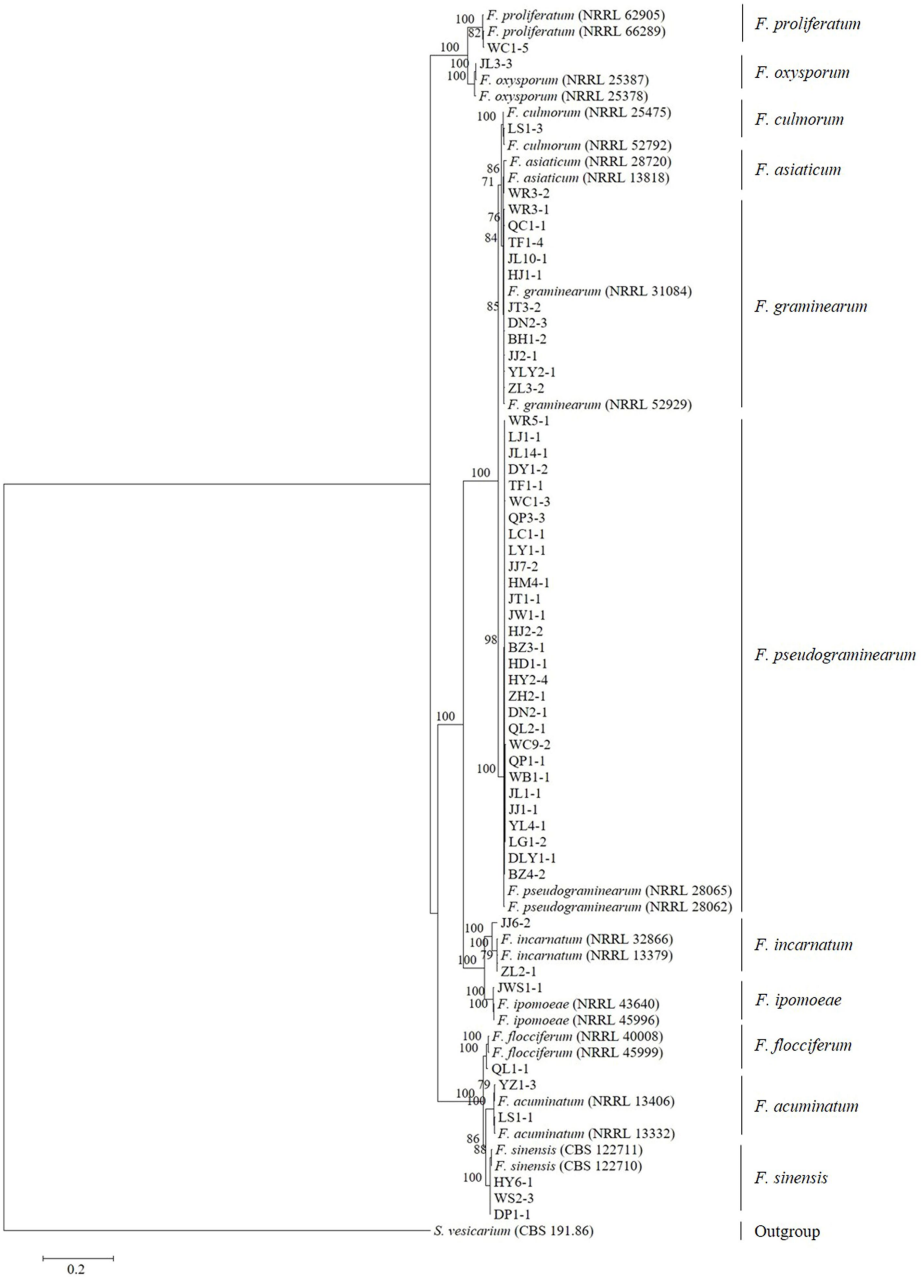
Maximum likelihood phylogenetic tree based on a concatenated alignment of *TEF-1α*, *RPB1*, and *RPB2* gene sequences. The tree was rooted using sequences of *Stemphylium vesicarium*. Support values at nodes represent RAxML bootstrap percentages with values ≥70% are shown above the branches.

Tree topology resulting from an ML analysis of the independent alignment of *TEF-1α* partial gene sequences divided the 53 representative isolates into 11 clades (*F. pseudograminearum*, *F. graminearum*, *F. sinensis*, *F. acuminatum*, *F. incarnatum*, *F. ipomoeae*, *F. flocciferum*, *F. proliferatum*, *F. asiaticum*, *F. culmorum*, and *F. oxysporum*) (Figure 2; Supplementary Figure S1), which is consistent with the result of BLASTn comparison. The phylogenetic tree based on the concatenated sequences of three loci (*TEF-1α*, *RPB1*, and *RPB2*) using the ML method divided the 53 representative isolates into 11 clades (Figure 3), which is congruent with the tree of independent data of the *TEF-1α* partial gene. These results indicated that using the *TEF-1α* partial gene to identify *Fusarium* species is rapid, effective, and accurate.

### Pathogenicity tests

**Figure 4:**
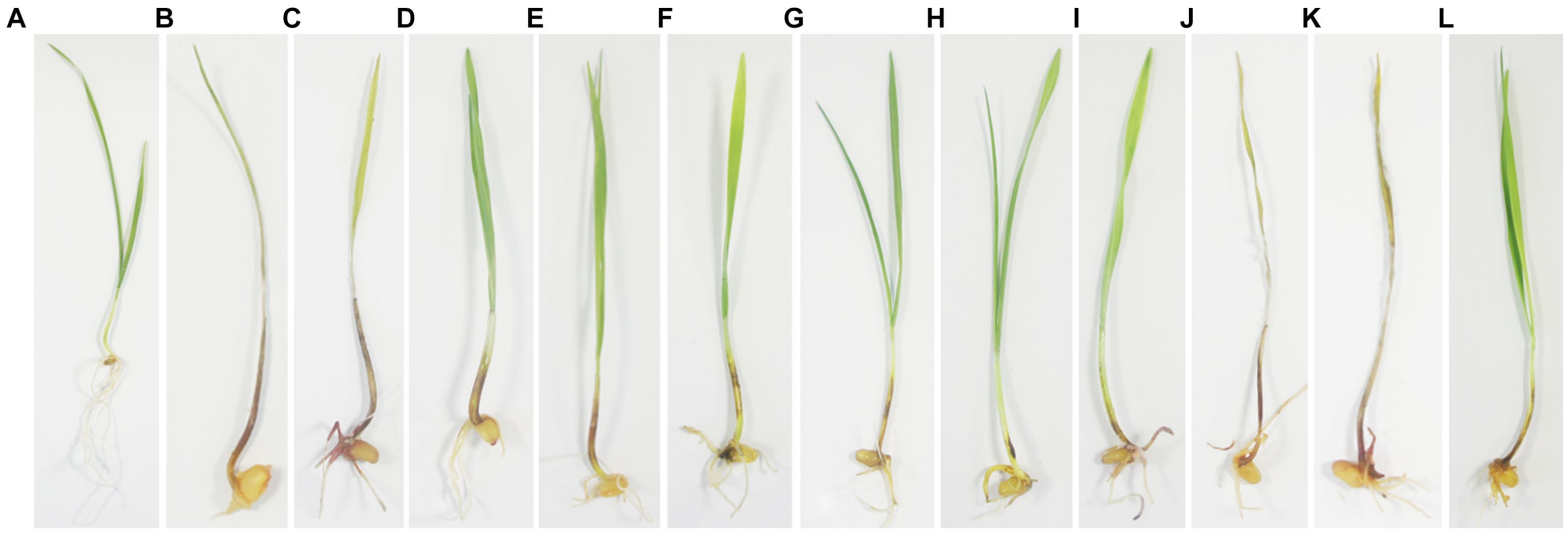
Pathogenicity assays of the representative isolates of 11 *Fusarium* species on wheat seedlings. **(A)** CK; **(B)** *Fusarium pseudograminearum*; **(C)** *F. graminearum*; **(D)** *F. sinensis*; **(E)** *F. acuminatum*; **(F)** *F. incarnatum*; **(G)** *F. ipomoeae*; **(H)** *F. flocciferum*; **(I)** *F. proliferatum*; **(J)** *F. asiaticum*; **(K)** *F. culmorum*; **(L)** *F. oxysporum*. The experiment was conducted on wheat seedlings ‘Jimai 22’. Agar plugs (5 mm in diameter) were placed on the base of wheat seedling stems, which were pregerminated at 28°C for 3 days. Disease severity (DS) was scored after 7 days of incubation at 25°C and 90% relative humidity using a six-point rating system.

The 418 tested *Fusarium* isolates, including 283\u00B0*F. pseudograminearum*, 75\u00B0*F. graminearum*, 24\u00B0*F. sinensis*, 12\u00B0*F. acuminatum*, 13\u00B0*F. incarnatum*, four *F. ipomoeae*, two *F. flocciferum*, two *F. proliferatum*, one *F. asiaticum*, one *F. culmorum*, and one *F. oxysporum*, could cause typical symptoms of FCR on wheat seedlings. The symptoms ranged from very faint lesions on the coleoptile only to intense brown necrotic discoloration on the leaf sheaths and finally to plant death resulting from stem rotting, while no symptoms of FCR were observed on control seedlings inoculated with PDA agar plugs not containing *Fusarium* mycelia (Figure 4). The average disease incidence and average disease index caused by *F. pseudograminearum*, *F. graminearum*, *F. sinensis*, *F. acuminatum*, *F. incarnatum*, *F. ipomoeae*, *F. flocciferum*, and *F. proliferatum* on wheat seedlings ranged from 38.3 to 99.1% and from 8.7 to 72.4, respectively. The disease incidence and disease index (98.1% and 72.4, 99.1% and 64.5, respectively) of *F. pseudograminearum* and *F. graminearum* were significantly higher than those of *F. sinensis* (64.2% and 15.6), *F. incarnatum* (74.9% and 17.9), and *F. ipomoeae* (60.8% and 12.8). Only one *F. asiaticum* isolate, one *F. culmorum* isolate, and one *F. oxysporum* isolate were identified among all 468 *Fusarium* isolates, and their disease incidence and disease index were 100.0% and 73.3, 100.0% and 76.7, and 100.0% and 26.0, respectively (Table 4). Isolates of *F. pseudograminearum*, *F. graminearum*, *F. asiaticum*, and *F. culmorum* generally exhibited a high level of virulence on wheat seedlings, while isolates of *F. sinensis*, *F. acuminatum*, *F. incarnatum*, *F. ipomoeae*, *F. flocciferum*, *F. proliferatum*, and *F. oxysporum* exhibited a relatively low level of virulence.

**Table 4:** Disease incidence and disease index of the 11 *Fusarium* species on wheat seedlings.

***Fusarium* species**	**Number of isolates**	**Disease incidence (%)**	**Disease index**
*F. pseudograminearum*	283	6.7 ~ 100.0 (98.1 ± 9.2) a	1.3 ~ 100.0 (72.4 ± 20.7) a
*F. graminearum*	75	36.7 ~ 100.0 (99.1 ± 7.3) a	7.3 ~ 90.0 (64.5 ± 11.2) b
*F. sinensis*	24	13.3 ~ 100.0 (64.2 ± 24.8) c	2.7 ~ 29.3 (15.6 ± 7.3) c
*F. acuminatum*	12	50.0 ~ 100.0 (92.2 ± 15.3) a	10.0 ~ 32.0 (25.6 ± 6.2) c
*F. incarnatum*	13	33.3 ~ 100.0 (74.9 ± 23.9) b	6.7 ~ 34.0 (17.9 ± 8.2) c
*F. ipomoeae*	4	40.0 ~ 76.7 (60.8 ± 15.5) c	8.0 ~ 16.0 (12.8 ± 3.7) c
*F. flocciferum* ^x^	2	36.7 ~ 40.0 (38.3 ± 2.4)	7.3 ~ 10.0 (8.7 ± 1.9)
*F. proliferatum* ^x^	2	63.3 ~ 90.0 (76.7 ± 18.9)	15.3 ~ 25.3 (20.3 ± 7.1)
*F. asiaticum* ^y^	1	100.0	73.3
*F. culmorum* ^y^	1	100.0	76.7
*F. oxysporum* ^y^	1	100.0	26.0

^x^Two isolates were used for pathogenicity tests of these two *Fusarium* species. ^y^Only one isolate was obtained for these three *Fusarium* species. So differences between *F. flocciferum*, *F. proliferatum*, *F. asiaticum*, *F. culmorum*, or *F. oxysporum* and other six *Fusarium* species could not be statistically analyzed. Agar plugs (5 mm in diameter) were inoculated at the base of wheat seedling stems, which were pregerminated at 28°C for 3 days. The wheat variety is ‘Jimai 22’. Disease severity (DS) was scored after 7 days incubation at 25°C and 90% relative humidity using a 6-point rating system. Values in parentheses are the mean ± standard deviation based on the data of each tested *Fusarium* isolate of the corresponding species. Values followed by different lowercase letters within a column are significantly different according to the least significant difference test (*p* < 0.05).

No *Fusarium* isolates were re-isolated from the control seedlings, while *Fusarium* isolates were consistently re-isolated from wheat seedlings with symptoms of FCR. The identities of the re-isolated fungi were confirmed by molecular characterizations as described above, thus fulfilling Koch’s postulates.

## Discussion

In this study, 11 *Fusarium* species were identified as causal agents of FCR in the main wheat-producing regions of Shandong province in China. The identified species were *F. pseudograminearum* (60.47%), *F. graminearum* (24.15%), *F. sinensis* (5.98%), *F. acuminatum* (3.85%), *F. incarnatum* (2.78%), *F. ipomoeae* (1.07%), *F. flocciferum* (0.64%), *F. proliferatum* (0.43%), *F. asiaticum* (0.21%), *F. culmorum* (0.21%), and *F. oxysporum* (0.21%). To our knowledge, this is the first report in the world of *F. incarnatum*, *F. ipomoeae*, and *F. flocciferum* causing crown rot of wheat.

A total of 468 *Fusarium* isolates were obtained from 199 wheat samples with FCR symptoms, and the isolation ratio of *Fusarium* species was 2.35 in the study. An earlier research from the Huanghuai wheat-growing region showed that 1,196 *Fusarium* isolates were isolated from 222 samples with the isolation ratio of 5.39 (Zhou et al., 2019). Another study showed that the isolation ratio of *Fusarium* species was 8.26, and the wheat samples were collected from central, eastern, and southeastern Kazakhstan (Bozoğlu et al., 2022).

This study revealed a change and diversity of *Fusarium* species that causes crown rot of wheat in Shandong province. A previous survey by Zhang et al. (2015) in the five major wheat-growing provinces of China, which include Shandong province, revealed that the dominant pathogen was *F. asiaticum*, followed by *F. graminearum*. Another study reported that *F. pseudograminearum*, *F. graminearum*, *F. sinensis*, *F. acuminatum*, *F. equiseti*, *F. proliferatum*, and *F. oxysporum* are the pathogens causing FCR in wheat in Shandong province, and *F. pseudograminearum* and *F. graminearum* are both the dominant pathogens and have the same isolation frequency (41%, respectively) (Zhou et al., 2019). Recent report indicated that *F. pseudograminearum*, *F. graminearum*, and *F. asiaticum* were responsible for crown rot of wheat in Shandong province, with *F. pseudograminearum* being the most prevalent species (Deng et al., 2020). Our results were consistent with those of previous studies, which showed that *F. pseudograminearum* was the dominant pathogen, but we found more abundant *Fusarium* species causing crown rot of wheat in the Shandong province, such as *F. incarnatum*, *F. ipomoeae*, *F. flocciferum*, and *F. culmorum*.

Climate may play a crucial role in determining the prevalence of *Fusarium* species. Temperature impacts the aggressiveness of *F. pseudograminearum*, while cooler diurnal temperatures (e.g., 15/15°C vs. 25/15°C) increased the aggressiveness of *F. pseudograminearum* (Sabburg et al., 2015). Deng et al. (2020) found that the frequency of *F. asiaticum* was higher than *F. graminearum* in Jiangsu province, while *F. asiaticum* was rarely isolated in Shandong province. The bias toward Jiangsu in the distribution of *F. asiaticum* coincided with the climate envelope modeling, indicating that *F. asiaticum* occurs in areas with warm and wet summers (Backhouse, 2014), as the year-round climate in Jiangsu is warmer and wetter than that of Shandong. Other reports highlighted that the distribution of *F. pseudograminearum* was related to low rainfall, raised temperatures in summer, or elevated levels of carbon dioxide (Melloy et al., 2010; Moya-Elizondo et al., 2011; Xu et al., 2018).

For the uniquely reported species, *F. incarnatum* was isolated from samples collected from Binzhou, Heze, Jining, and Zibo (the inland areas) and Qingdao and Yantai (the coastal areas), *F. ipomoeae* was isolated from Jining and Weifang (the inland areas), and *F. flocciferum* was isolated from Heze and Qingdao. Climatic differences may not affect the distribution of *F. incarnatum*, *F. ipomoeae*, and *F. flocciferum* since *F. incarnatum* and *F. flocciferum* were found in the inland areas and the coastal areas, respectively, and *F. ipomoeae* was reported to be the pathogen of peanut leaf spot in Laixi (the coastal areas), China (Xu et al., 2021) and the pathogen of soybean wilt in South Korea (Choi et al., 2023). Naeem et al. (2019) considered that the diversity of *Fusarium* species on intercropped soybean pods was associated with soybean varieties. Further studies are needed to confirm whether the wheat variety affects the distribution of the three uniquely reported species.

The results of the assessment of pathogenicity show that all *Fusarium* isolates tested for pathogenicity could cause symptoms of FCR. *F. culmorum* was the most virulent species, followed by *F. asiaticum*, *F. pseudograminearum*, *F. graminearum*, *F. oxysporum*, *F. acuminatum*, *F. proliferatum*, *F. incarnatum*, *F. sinensis*, *F. ipomoeae*, and *F. flocciferum*. However, *F. culmorum* and *F. asiaticum* had low isolation percentages (each was 0.21%) and were only recovered from the cities of Liaocheng and Weihai, respectively. In contrast, the most prevalent species, *F. pseudograminearum*, was isolated from the samples collected in all sampled cities. Similarly, *F. graminearum* was commonly isolated except from the samples collected in Linyi city. Therefore, *F. pseudograminearum* and *F. graminearum* should be regarded as the major pathogens when designing and implementing disease management programs.

Maize [*Zea mays* L.] is an important food and feed crop and often rotated with wheat in Shandong province. Maize seedling blight commonly occurred in the Shandong province, and the disease incidence was up to 50% in some fields. The root system of infected plants displayed poor development. The primary roots were brown and rotted. The leaves at the base of the plants were drying up, and then, the whole plant withered (Jiang et al., 2022). Maize seedling blight is a serious threat to maize yield. Recently, it was first reported that *F. pseudograminearum* caused maize seedling blight in Zibo city, Shandong province (Jiang et al., 2022), which indicated that the crown rot of wheat caused by *F. pseudograminearum* may aggravate the occurrence of maize seedling blight. Controlling the occurrence of FCR and changing rotation crops are particularly important for the healthy production of wheat and maize.

Fusarium head blight (FHB) is a devastating disease affecting wheat in many regions throughout the world. It has the capacity to destroy a potentially high-yielding crop within a few weeks of harvest (McMullen et al., 1997). In addition to direct yield losses, FHB reduces grain quality, and the harvested grain is often contaminated with mycotoxins (Seitz et al., 1986). Previous studies reported that *F. pseudograminearum* and *F. graminearum* are also the major pathogens of FHB (Xu et al., 2015, 2021). The relationship between FCR and FHB needs further study. As FCR of wheat caused by *F. pseudograminearum* is an increasing problem in the Shandong province, it is appropriate to monitor the role of *F. pseudograminearum* in FHB in the future.

The use of clean and chemically disinfected seeds, adjusting the date of seeding, proper fertilization, crop rotations avoiding other host crops, and use of cultivars with resistance to the pathogens or to water stress have been suggested for the management of FCR of wheat (Cook, 2010). Among these strategies, fungicide seed treatment has always been a primary method for controlling FCR (Moya-Elizondo and Jacobsen, 2016). The accurate identification of *Fusarium* species is critical to disease management. Among *F. avenaceum*, *F. culmorum*, *F. graminearum*, and *F. poae*, it was observed that *F. graminearum* showed the highest sensitivity to prochloraz and *F. poae* showed lower sensitivity to metconazole compared to *F. culmorum* (Tini et al., 2020). As *F. pseudograminearum* and *F. graminearum* were confirmed as the causal agents of FCR of wheat in the Shandong province, further research should focus on the sensitivity of these two *Fusarium* species to commonly used fungicides.

## Data availability statement

The data presented in the study are deposited in the GenBank repository. The accession numbers can be found in the article.

## Author contributions

GM: Conceptualization, Funding acquisition, Writing – original draft, Writing – review & editing. HW: Investigation, Resources, Writing – review & editing. KQ: Resources, Writing – review & editing. LM: Formal analysis, Writing – review & editing. BZ: Methodology, Writing – review & editing. YZ: Writing – review & editing. HJ: Writing – review & editing. XW: Writing – review & editing. JQ: Funding acquisition, Project administration, Writing – review & editing.

## Supplementary material

Supplementary Figure S1 Maximum likelihood phylogenetic analysis of 11 Fusarium species based on *TEF-1α* partial gene sequences. Two strains of *F. solani* (NRRL 23244 and 32810) are the outgroup. The number of bootstrap replications was set to 1000. Support values at nodes represent bootstrap percentages with values ≥ 70% are shown above the branches.
