# Supplementary material for: Isolation, characterization, and pathogenicity of Fusarium species causing crown rot of wheat
Source: Front Microbiol. 2024 May 30;15:1405115. doi: 10.3389/fmicb.2024.1405115 (PMC11169711; doi:10.3389/fmicb.2024.1405115)
Supplement: Supplementary Figure S1 — Maximum likelihood phylogenetic analysis of 11 Fusarium species based on TEF-1α partial gene sequences. Two strains of F. solani (NRRL 23244 and 32810) are the outgroup. The number of bootstrap replications was set to 1000. Support values at nodes represent bootstrap percentages with values ≥ 70% are shown above the branches. [file Data_Sheet_1.pdf]

Table S1. Prevalence of individual *Fusarium* species or combinations recovered from diseased wheat samples.

Species[a]	Numbers of samples	Percentage of samples with species present	Total percentage
Fpg	87	43.72%	83.42%
Fg	46	23.12%	
Fsi	18	9.05%	
Fac	6	3.02%	
Fi	5	2.51%	
Fip	1	0.50%	
Ff	1	0.50%	
Fas	1	0.50%	
Fc	1	0.50%	
Fpg+Fg	14	7.04%	15.58%
Fpg+Fsi	1	0.50%	
Fpg+Fac	3	1.51%	
Fpg+Fip	1	0.50%	
Fpg+Fpr	1	0.50%	
Fg+Fsi	1	0.50%	
Fg+Fac	1	0.50%	
Fg+Fi	4	2.02%	
Fg+Fpr	1	0.50%	
Fsi+Fac	2	1.01%	
Fsi+Ff	1	0.50%	
Fi+ Fip	1	0.50%	
Fpg+Fg+Fi	1	0.50%	1.00%
Fpg+Fi+Fox	1	0.50%	

[a]: Fpg (*F. pseudograminearum*), Fg (*F. graminearum*), Fsi (*F. sinensis*), Fac (*F. acuminatum*), Fi (*F. incarnatum*), Fip (*F. ipomoeae*), Ff (*F. flocciferum*), Fpr (*F. proliferatum*), Fas (*F. asiaticum*), Fc (*F. culmorum*), Fox (*F. oxysporum*).
