# Supplementary figures and images for: Isolation, characterization, and pathogenicity of Fusarium species causing crown rot of wheat
Source: Front Microbiol. 2024 May 30;15:1405115. doi: 10.3389/fmicb.2024.1405115 (PMC11169711; doi:10.3389/fmicb.2024.1405115)

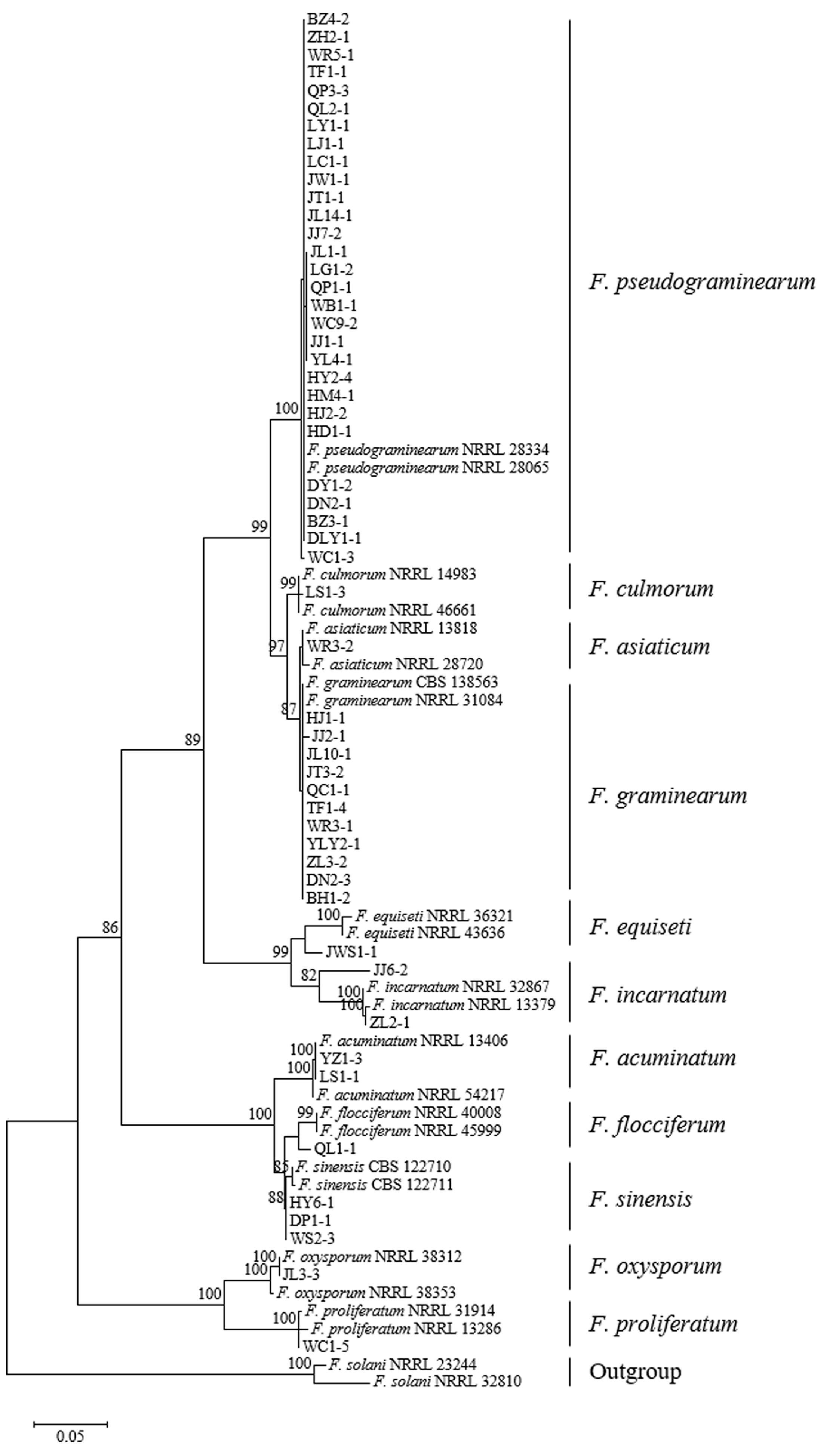

Supplement: Supplementary file 2 [file Image_1.TIF]
